# Case Report: Long-term follow-up of desert hedgehog variant caused 46, XY gonadal dysgenesis with multiple complications in a Chinese child

**DOI:** 10.3389/fgene.2022.954288

**Published:** 2022-08-22

**Authors:** Lili Pan, Zhuoguang Li, Zhe Su, Wei Su, Rongfei Zheng, Weiyan Chen, Xuezhi He, Jianming Song, Shoulin Li, Pengqiang Wen

**Affiliations:** ^1^ Department of Endocrinology, Shenzhen Children’s Hospital, Shenzhen, China; ^2^ Department of Neural Electrophysiology, Shenzhen Children’s Hospital, Shenzhen, China; ^3^ Department of Ultrasonography, Shenzhen Children’s Hospital, Shenzhen, China; ^4^ Department of Pathology, Shenzhen Children’s Hospital, Shenzhen, China; ^5^ Department of Urology, Shenzhen Children’s Hospital, Shenzhen, China; ^6^ Shenzhen Institute of Pediatrics, Shenzhen Children’s Hospital, Shenzhen, China

**Keywords:** DHH, Hedgehog signaling pathway, 46, XY gonadal dysgenesis, neuropathy, complications

## Abstract

**Background:** Desert hedgehog (DHH), as a member of the Hedgehog (HH) family, is mainly involved in testicular development and peripheral nerve sheath formation. A *DHH* variant has been identified in patients with 46, XY gonadal dysgenesis (46, XY GD) with or without neuropathy, but few reports mention the involvement of other complications.

**Case presentation:** Here, we report a Chinese female patient who was hospitalized at 14.3 years old due to slow breast development for more than 1 year. She had a female genitalia phenotype and breast development started at 13 years old but progressed slowly. She was not yet menarche on admission, and she had intermittent muscle cramps in her hands and feet. Her karyotype analysis was 46, XY and the *SRY* gene was positive. Surgical exploration revealed no uterus or ovaries, and the pathology of bilateral gonads was dysplastic testis tissue, which was consistent with partial gonadal dysgenesis (PGD). Genetic analysis identified a homozygous pathogenic variant in *DHH* exon 3 (c.1027T>C, p. Cys343Arg). During the 6-year follow-up, she received estrogen replacement therapy, resulting in breast development progression without gender dysphoria. However, her peripheral neuropathy became more obvious, and a nerve conduction study (NCS) indicated decreased nerve conduction velocity and action potential. In addition, she also suffered complications such as obesity, insulin resistance, fatty liver, and gastric ulcers.

**Conclusion:** In the present study, we reported a case of 46, XY GD with minifascicular neuropathy caused by a *DHH* homozygous variant, and we summarized the reported cases worldwide. For the first time in such patients, we showed a comparison of NCS changes with age as well as the presence of multiple complications not previously reported.

## Introduction

Desert hedgehog (*DHH*, OMIM 605423), a member of the Hedgehog (HH) family, is homologous to the Sonic HH and Indian HH families in mammals. The HH signaling pathway regulates embryonic development, postnatal cell proliferation, and differentiation, which are related to the development and tissue homeostasis of nerves, bones, gonads, gastrointestinal, liver, and other organs. Among them, *DHH* is mainly involved in testicular development and peripheral nerve sheath formation (Bitgood et al., 1996; Boso et al., 2020; Qi et al., 2021). In 2000, Umehara (Umehara et al., 2000) first identified the *DHH* gene variant in patients with 46, XY gonadal dysgenesis (46, XY GD), also known as 46, XY disorder of sex development (46, XY DSD). As an extremely rare autosomal recessive disease, only 25 cases in detail have been reported worldwide so far, and the clinical manifestations mainly include delayed development of secondary sexual characteristics, amenorrhea, and abnormal external genitalia with or without peripheral neuropathy (Sato et al., 2017). There are fewer cases of the disease in childhood, and long-term follow-up and other systemic involvement are rarely reported. Here, we reported a Chinese child with a homozygous missense variant in the *DHH* gene, and during the 6-year follow-up, in addition to the description of the gonads, we showed a comparison of pre- and post-NCS changes with age as well as the presence of multiple complications not previously reported. Our report will expand the phenotypic spectrum of diseases caused by the *DHH* variant, and we suggest that these patients require long-term follow-up and comprehensive management.

## Case presentation

### General information

Our patient was a 14.3-year-old female (social gender) of Han nationality from Hainan, China. She was admitted to the Department of Endocrinology, Shenzhen Childrens Hospital due to slow breast development for more than 1 year. At 13 years old, breast development started but progressed slowly, accompanied by the growth of axillary and pubic hair, and she had a height increase of approximately 10 cm/year. In addition, she had intermittent muscle spasms in the hands and feet, which were typically mild and short-lived at first, and they were gradually relieved without intervention. She performed poorly in sports, such as throwing, running, and jumping, but she had good academic performance and normal intellectual development.

Personal history and family history: She was born to a G1P1 healthy mother *via* vaginal delivery at full-term. At 13 years old, she went to the psychiatry department due to depression; she was diagnosed with children’s psychological behavior disorder and her mood improved after escitalopram oxalate administration. She denied a family history of gonadal dysgenesis, and her parents were from two unrelated families. Her father’s height was 173 cm and her mother’s height was 156 cm, which indicated that her genetic potential height was 158 cm (−0.4 SD).

### Physical examination

Her height, weight, and BMI were 165.0 cm (+1.1 SD), 55.9 kg (+0.9 SD), and 18.9 kg/m^2^ (+0.1 SD), respectively. She had a normal appearance without special features. Her bilateral breasts were in Tanner stage B3 with normal areola color, and her bilateral axillary hair was in stage A2. No mass was palpable in the inguinal area, and her pubic hair was in stage PH3. She had a typical female vulva without clitoral hypertrophy, and two urogenital sinus openings were visible. The rest of the physical examination was basically normal.

### Auxiliary examination

Sexual development-related hormone examination: A GnRH stimulation test suggested hypergonadotropic hypogonadism (basal luteinizing hormone (LH) 40.94 IU/L, peak LH>250 IU/L; basal follicle-stimulating hormone (FSH) 121.94 IU/L, peak FSH>208 IU/L), and the HCG stimulation test suggested primary hypogonadism (no testosterone elevation: 2.97 to 2.91 nmol/L). The anti-Müllerian hormone (AMH) level was 1.0 μg/L, and the inhibin B level was 17.52 ng/L. Her karyotype analysis showed 46, XY and the *SRY* gene was positive. Other laboratory investigations revealed that there were no abnormalities in the hepatic/renal function, serum glucose, serum lipids, thyroid function, and tumor markers.

Pelvic ultrasonography revealed heterogeneous hypoechoic masses in the upper segment of the bilateral inguinal canals with sizes of 1.2 × 0.7 × 0.6 cm and 2.3 × 0.9 × 1.0 cm, and no uterine or ovarian echoes were observed. According to the GP method, her bone age was 13.5 years old and the height for bone age was +1.6 SD. NCS (at 15.4 years old) showed that the motor and sensory nerve conduction velocity of the detected nerves, and the sensory nerve action potential (SNAP) of the sural nerves were slightly decreased, and the F–M latency of the tibial nerves were mildly prolonged (see Table 1).

**TABLE 1:** Comparison of NCS results during the follow-up of the present patient.

Age (years)	Median nerves (left/right)			Tibial nerves (left/right)		Sural nerves (left/right)		F–M latency (ms)
	MCV (m/s)	SCV (m/s)	CMAP (mV)	MCV (m/s)	CMAP (mV)	SCV (m/s)	SNAP (mV)	
15.4	49.1/49.3	44.4/46.0	13.9/17.3	39.0/34.7	7.2/5.8	39.1/39.7	7.9/5.2	54.7/56.6
18.4	48.6/46.1	43.1/42.3	15.5/16.0	34.9/35.5	5.1/5.0	38.5/36.2	4.2/2.4	55.1/-
Reference mean value	62.0 ± 5.3	60.6	10.5 ± 3.68	45.5 ± 3.8	14.92 ± 3.37	52.1 ± 5.1	8.67 ± 2.12	47.4 ± 3.3

MCV, motor nerve conduction velocity; SCV, sensory never conduction velocity; CMAP, compound motor action potential; SNAP, sensory nerve action potential.

Gender dysphoria questionnaire (female): She was assessed using the adolescent gender dysphoria questionnaire, but she did not meet the corresponding symptoms, suggesting that she didn't have gender dysphoria.

Genetic analysis: After obtaining informed consent, genetic analysis of the proband and her parents was performed by Guangzhou KingMed Medical Diagnostics Center. Next-generation sequencing was utilized to sequence the exon coding regions of 64 DSD-related genes (see Supplementary Table S1). The candidate gene was also verified by Sanger sequencing and parental sequencing. Finally, we identified a homozygous variant in the *DHH* exon 3 (c.1027T>C, p. Cys343Arg), and a heterozygous variant was found at this site in both her parents (see Supplementary Table S1). According to the 2015 ACMG guidelines, this missense variant was pathogenic.

### Surgical exploration and gonadal pathology

**FIGURE 1:**
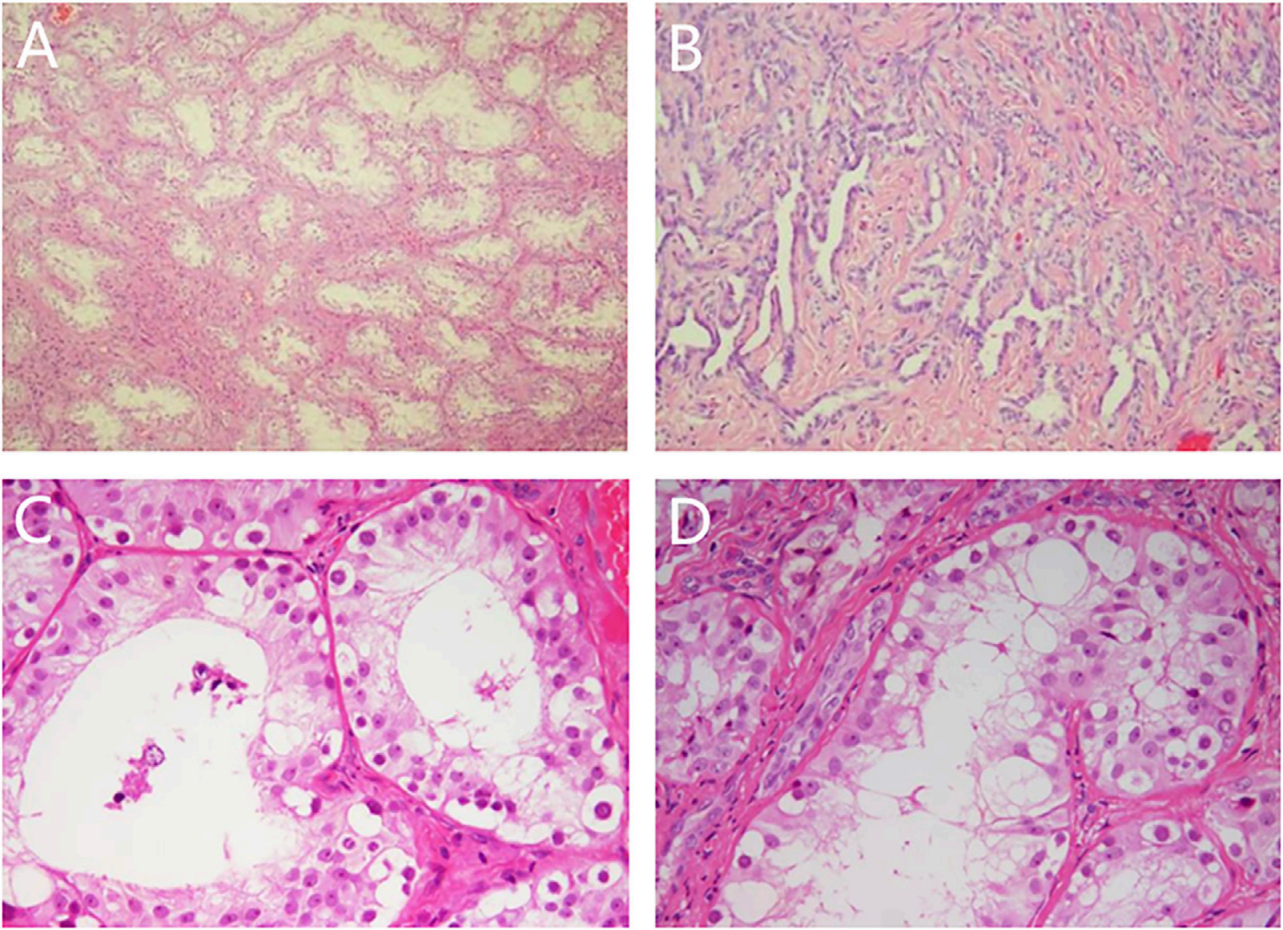
Pathological results of bilateral gonads. Note: **(A)** (HE × 50) and **(C)** (HE × 200): left gonad, spermatogonia could be seen in seminiferous tubules; **(B)** (HE × 100) and **(D)** (HE × 200): right gonad, adenoid hyperplasia of testicular hilar cells could be seen.

Laparoscopic exploration: The bilateral gonads were located in the abdominal cavity, and no uterus, ovaries, or fallopian tubes were found in the pelvic cavity. Cystoscopy showed that there was a blind-end vagina approximately 3 cm from the opening. The bilateral gonads were removed laparoscopically, and the pathology of the bilateral gonads was dysplastic testis tissue (see Figure 1), consistent with PGD, and no tumor cells were found.

### Treatment and follow-up

During the 6-year follow-up, her personality as a female was more obvious and she loved to dress-up without gender dysphoria. She continued to receive low-dose estrogen replacement therapy and the dose was gradually increased, and her breast development progressed from Tanner stage B3 to B5. However, the muscle spasm in her hands and feet were worse than than before, and there was an episode in her cheeks, which was more obvious after tiredness and stress. At a follow-up 3 years ago, she suffered upper abdominal discomfort, and a gastroscopic examination revealed gastric ulcers. After 2 weeks of oral medication, her symptoms improved but recurred after she discontinued the medication.

**FIGURE 2:**
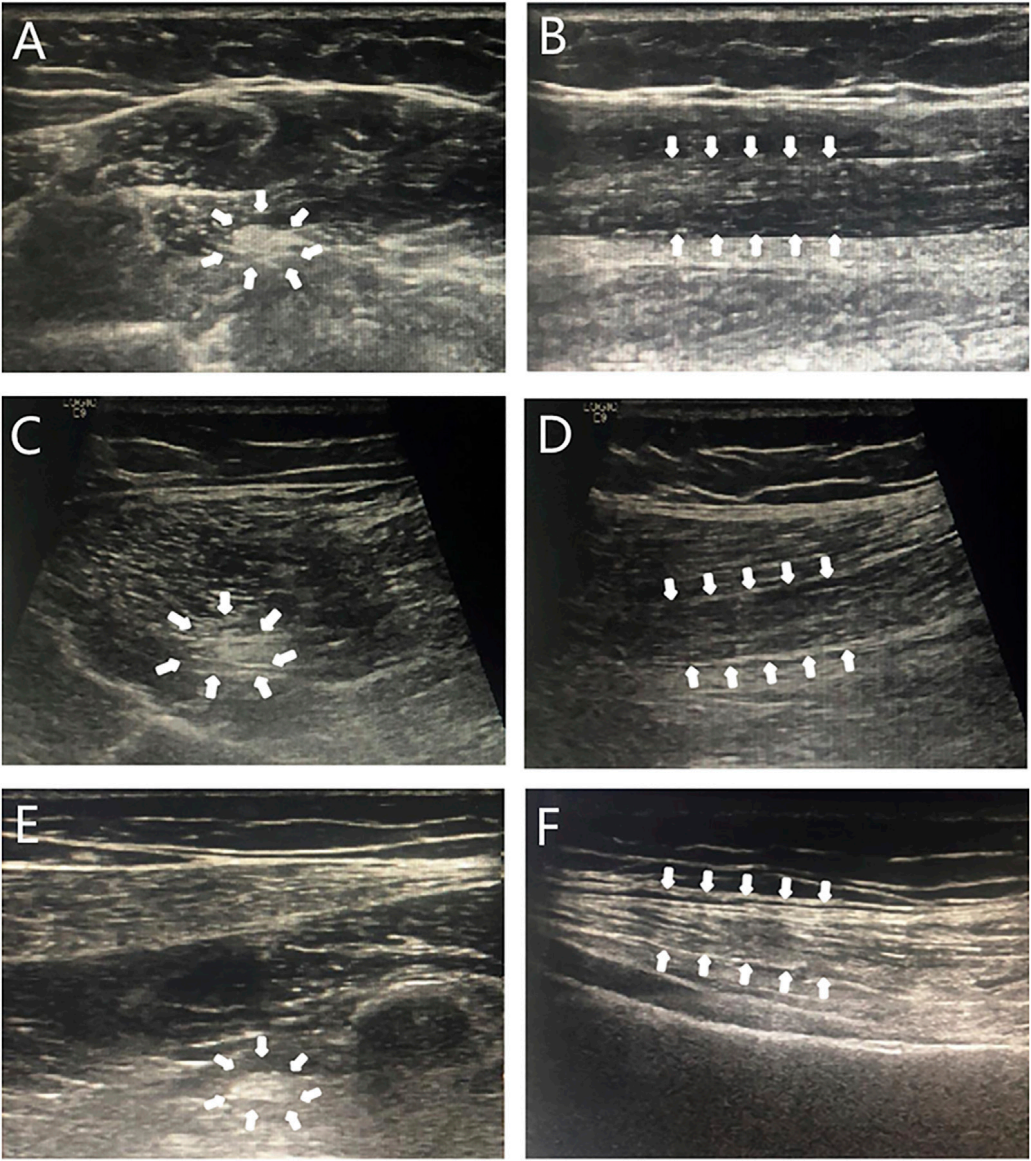
Sonogram of neurosonography. Note: Transverse sections **(A,C,E)**: punctate hyperechoic; longitudinal sections **(B,D,F)**: diffuse hyperechoic, less clear texture. A and B: median nerve; C and D: tibial nerve; E and F: peroneal nerve; arrows: the area shown was an abnormal signal on neurosonography.

During the last examination in our hospital (at 18.4 years old), her height, weight, and BMI were 175.5 cm (+2.8 SD), 76.8 kg (+2.7 SD), and 24.9 kg/m^2^ (24-28 kg/m^2^ for overweight), respectively. Her bilateral breasts, axillary hair, and pubic hair were in stage B5, stage A3, and stage PH4, respectively. Evaluation of her glucose metabolism indicated fasting serum glucose, fasting insulin, and serum C-peptide and HOMA-IR levels of 4.84 mmol/L, 18.5 mIU/L, 2.84 μg/L, and 3.98, respectively, suggesting insulin resistance. Although no abnormality was found in her hepatic/renal function and serum lipids, liver ultrasonography indicated the presence of nonalcoholic fatty liver disease. A body composition examination was performed with the following results: percentage of body fat of 42.5% (reference range 18%–28%), the muscle index of 31% (reference range 33%–43%), and the area of visceral fat of 171.3%. No abnormal discharge waves were found in an electroencephalogram (EEG) examination. Re-examination of NCS showed that nerve conduction velocities and action potential were decreased, and that the FM wave latency was longer than before (see Table 1). High-resolution neurosonography showed changes in peripheral nerves, which were manifested as increased echogenicity of the median, tibial, and superficial peroneal nerve (see Figure 2). The gender dysphoria questionnaire indicated no gender dysphoria.

Therapy adjustment: Vitamin B was recommended for peripheral nerve nutrition, and metformin treatment and health education were provided for obesity as well as insulin resistance. According to a recent telephone follow-up (20 years old), her weight gain was under control, but her muscle cramps were still obvious and had not improved by vitamin B therapy.

### Literature review

Relevant studies were searched from PubMed, Web of Science, HGMD, and some Chinese databases, such as CNKI, Wanfang, and VIP Database, up to May 2022. Our searches were based on combinations of the following index terms: *DHH*, 46, XY gonadal dysplasia, 46, XY gonadal dysgenesis, 46, XY disorder of sex development, neuropathy, Hedgehog signaling pathway and the corresponding terms in Chinese. A total of 25 cases of *DHH* variants causing 46, XY GD in detail have been reported worldwide so far (Umehara et al., 2000; Canto et al., 2004; Das et al., 2011; Werner et al., 2015; Paris et al., 2017; Sato et al., 2017; Baldinotti et al., 2018; Rothacker et al., 2018; Tajouri et al., 2018; Ayers et al., 2019; Buonocore et al., 2019; Neocleous et al., 2019; Akter et al., 2021; Kalinchenko et al., 2021; Mehta et al., 2021) (see Table 2), including 24 different variant types of missense, deletion, duplication, or transversion, and both homozygous and compound heterozygous variants have been reported. The reported ages ranged from 2.8 to 55 years. Moreover, 23 cases were raised as females and two cases were raised as males. These patients were mainly referred for delayed development of secondary sexual characteristics, amenorrhea, or abnormal external genitalia. There were 19 patients with homozygous variants, and their gender was female with a complete female phenotype. The gonadal pathology could be complete gonadal dysgenesis (CGD) or PGD. Among these patients, nine cases had neuropathy, except for three patients with incomplete information, and the incidence of neuropathy was 56% (9/16). There were six patients with compound heterozygous variants, including four females and two males of social gender, and none of them had neuropathy. The pathological results of gonads were reported in 19 cases, including nine cases of PGD and 10 cases of CGD, and 25% (4/16, three cases not mentioned) cases of them had gonad tumors. Obesity, insulin resistance, fatty liver, and gastric ulcers were not reported in the above literature.

**TABLE 2:** Clinical features of reported patients with *DHH* variant caused 46, XY gonadal dysgenesis.

Author (time)	*DHH* variant	Variation type	Age (years)	Social gender	Chief complaint	Neuropathy	Gonadal pathology	Gonad tumors	Müllerian duct structure
Umehara et al. (2000)	c.2T>C	Hom	27	Female	Amenorrhea	+	PGD	–	Naive uterus
Canto et al. (2004)	c.485T>C	Hom	16	Female	NM	NM	CGD	–	Naive uterus
	c.1086delG	Hom	19	Female	NM	NM	CGD	Gonadoblastoma	Naive uterus
	c.1086delG	Hom	26	Female	NM	NM	CGD	Dysgerminoma	Naive uterus
Das et al. (2011)	c.57_60dupAGCC	Hom	17	Female	Amenorrhea	–	CGD	–	Naive uterus
	c.271_273delGAG	Hom	26	Female	Amenorrhea	–	CGD	–	Naive uterus
Werner et al. (2015)	c.371G>A	Hom	23	Female	Amenorrhea	+	PGD	Gonadoblastoma, seminoma	Blind-end vagina, no uterus
	c.371G>A	Hom	30	Female	Amenorrhea	+	PGD	Seminoma	Blind-end vagina, no uterus
Paris et al. (2017)	c.519G>T	Hom	26	Female	Amenorrhea	+	NM	NM	–
	c.519G>T	Hom	23	Female	Amenorrhea	–	NM	NM	NM
Sato et al. (2017)	c.304-572_492dup	Hom	55	Female	Amenorrhea	+	CGD	–	Naive uterus
	c.304-572_492dup	Hom	47	Female	Amenorrhea	+	CGD	–	Naive uterus
Baldinotti et al. (2018)	c.554C>A	Hom	54	Female	Amenorrhea	+	PGD	–	–
Rothacker et al. (2018)	c.491G>C	Hom	14	Female	Amenorrhea	–	PGD	–	–
Tajouri et al. (2018)	c.528C>A,	cHet	21	Female	Amenorrhea	–	NM	NM	NM
	c.1011delC								
	c.528C>A,	cHet	2.8	Male	AEG	–	NM	NM	NM
	c.634G>A								
Ayers et al. (2019)	c.782C>T, c.680C>T	cHet	NM	Female	AEG	–	PGD	–	Blind-end vagina, no uterus
	c.746G>A, c.508G>A	cHet	NM	Male	AEG	–	NM	NM	–
Buonocore et al. (2019)	c.724T>C	Hom	Adult	Female	Amenorrhea	–	CGD	NM	–
	c.734G>C, c.680C>T	cHet	Adult	Female	AEG	–	PGD	NM	–
Neocleous et al. (2019)	c.491G>C	Hom	19	Female	Amenorrhea	–	CGD	–	–
Akter et al. (2021)	c.863G>C	Hom	22	Female	DDSSC	–	NM	NM	–
Kalinchenko et al. (2021)	c.419T>G	Hom	14	Female	DDSSC	+	PGD	–	Blind-end vagina, no uterus
	c.419T>G	Hom	14	Female	DDSSC	+	PGD	–	Blind-end vagina, no uterus
Mehta et al. (2021)	c.1156insG,	cHet	Adult	Female	Amenorrhea, poorly developed breasts	NM	CGD	NM	NM
	c.997A>G								
This case	c.1027T>C	Hom	14.3	Female	DDSSC	+	PGD	–	Blind-end vagina, no uterus

+, Positive, existed after evaluation; –: Negative, did not exist after evaluation; NM, not mentioned; Hom, homozygous; cHet, compound heterozygotes; AEG, abnormal external genitalia; DDSSC, delayed development of secondary sexual characteristics; PGD, partial gonadal dysgenesis; CGD, complete gonadal dysgenesis.

## Discussion

*DHH* gene variants have been identified in patients with 46, XY GD, manifesting as a female phenotype or incomplete masculinization. More than half of the patients with homozygous variants suffer peripheral neuropathy, which is the so-called 46, XY GDMN (OMIM 607080). Neuropathy in these patients may gradually worsen with age, and even lead to digital ulcers and amputation in severe cases. The gonadal pathology of patients with homozygous variants may be CGD or PDG, with the former being predominant. The phenotype of patients with compound heterozygous variants is relatively mild, and in addition to the absence of peripheral neuropathy, the difference is also manifested in gonadal function; these patients may still have a certain degree of virilization and their gonadal pathology is PGD (Tajouri et al., 2018; Ayers et al., 2019; Buonocore et al., 2019). Ayers and Elzaiat (Ayers et al., 2019; Elzaiat et al., 2020) suggested that the *DHH* homozygous variant affects the overall conformation of the protein, thereby perturbing its self-cleavage, resulting in a significant loss (1%) of functional activity compared to the wild type, and they reported that compound heterozygotes show a significant decrease in activity (2% and 46%) and that single heterozygotes have nearly wild-type activity (90% and 98%). Similar to this case, patients with homozygous variants of *DHH* are more severe than those with compound heterozygous variants.

DHH belongs to the highly conserved HH protein family. The HH cell signaling pathway molecule was first discovered in *Drosophila* in 1980; it is involved in cell proliferation, cell differentiation, and maintenance of tissue homeostasis (Chai et al., 2021). DHH is an important regulator of gonadal development and is specifically expressed in the testis, but not in the ovary. The *dhh* ^−/−^ male animal model has a female phenotype with hypogonadism, decreased Leydig cell number, lack of mature spermatogenesis, incomplete testicular descent, and the existence of a blind-end vagina, suggesting that *DHH* is involved in the development, descent, and spermatogenesis of the testis (Bitgood et al., 1996; Yao et al., 2002; Kawai et al., 2011; Qi et al., 2021). After the expression of *SRY* in the early embryonic stage, DHH is secreted by Sertoli cells and acts on fetal Leydig cells (FLCs) and peritubular myoid cells in a paracrine manner. The functions of FLCs involve the synthesis and secretion of androgen, thereby promoting the development of the male reproductive system, while peritubular myoid cells directly or indirectly regulate the function of Leydig cells through paracrine factors of Sertoli cells (Bitgood et al., 1996; Martin, 2016; Chen et al., 2017). A *DHH* variant inhibits FLC proliferation and differentiation as well as affects the differentiation of peritubular myoid cells and formation of the testicular cord, thus causing testicular dysplasia. In addition, DHH can also act on Sertoli cells in an autocrine manner. The absence of uteruses in *DHH*-mutated cases with incomplete masculinization (Ayers et al., 2019; Buonocore et al., 2019) has suggested that Sertoli cells in early embryos may still secrete AMH, thereby inhibiting the development of the Müllerian duct structure; however, due to the persistent abnormality of gonad position and autocrine function, the function of Sertoli cells is reduced to varying degrees causing spermatogenesis disorders in patients. In contrast, naive uteruses have been observed in patients with the female phenotype, suggesting severely impaired AMH secretion in early embryonic stages. In the present case, the basal FSH was significantly increased, and testosterone did not respond after hCG stimulation. Moreover, the pathology showed testicular dysplasia, while AMH was low (a small amount of secretion could still be detected). Thus, Sertoli cells were relatively less dysfunctional than significantly increased FSH levels and severely dysfunctional Leydig cells, indicating that Sertoli cells developed better than Leydig cells during the embryonic stage of *DHH* variant patients. In some patients, CGD or PGD due to a *DHH* variant increases the risk of germ cell tumors, which develop into seminoma, dysgerminoma, and gonadoblastoma (Canto et al., 2004; Werner et al., 2015), with an incidence of 25%. Therefore, patients with a *DHH* variant should be closely monitored, and their dysplastic gonads should be removed if necessary.

*DHH* is also highly expressed in Schwann cells and participates in the formation of nerve sheaths, which is necessary for the integrity and normal function of peripheral nerves. Electrophysiological studies in *dhh* ^−/−^ mice have shown that nerve fiber conduction velocity is significantly decreased, which is related to axon reduction, increased nerve sheath–vascular permeability, and nerve fiber degeneration (Sharghi–Namini et al., 2006; Sato et al., 2017; Boso et al., 2020). Peripheral neuropathy in more than 50% of patients with *DHH* homozygous variants may cause sensory and motor nerve disorders, mainly the former, manifesting as spasticity, numbness, or weakness of hands and feet. Electrophysiological examination has shown decreased conduction velocity and prolonged F–M latency, and high-resolution neurosonography has shown diffuse hyperechoic in the transverse and longitudinal sections of the nerve bundle (Werner et al., 2015; Boso et al., 2020). GDMN usually appears in adult patients aged 20–30 years, but some children with GDMN have been reported in recent years (Sato et al., 2017; Kalinchenko et al., 2021). The present patient had atypical onset symptoms, which may have been related to the young age, but the comparison of her previous and recent NCS examinations showed that it was worsening after only 6 years. Because the symptoms of peripheral neuropathy worsen with age, long-term follow-up and monitoring should be performed for early detection and prevention.

In addition, some studies (Pospisilik et al., 2010; Matz-Soja et al., 2016; Braune et al., 2017; Guillen–Sacoto et al., 2017) have suggested that with the weakening of HH pathway signaling, there may be increased adipose tissue inflammation, impaired glucose tolerance, induction of hepatic steatosis, and impaired gastric cell regeneration and homeostasis. The HH molecules have various functions as follows: DHH and IHH regulate glucose metabolism and adipose tissue differentiation, SHH and IHH are involved in hepatocyte repair and lipid metabolism, and SHH is related to gastric cell regeneration (Kang et al., 2009). A previous study on an obese population (Braune et al., 2017) has reported that DHH and IHH are expressed in visceral and subcutaneous adipose tissue. With the increase in BMI, the ligands of the HH signaling pathway decrease, and the expression of DHH is significantly negatively correlated with obesity-related indicators (such as body weight, waist circumference, hip circumference, body mass percentage, and fat area). Knockout of the SMO receptor in the HH signaling pathway increases body weight, increases adipose tissue inflammation, and impairs glucose tolerance. The BMI of the patient in our study was only 24.9 kg/m^2^, but the percentages of body mass and fat area were significantly increased, which may be related to the abnormal expression of the DHH protein affecting the normal conduction of the HH signaling pathway. During the follow-up, she gradually developed obesity, insulin resistance, fatty liver, and gastric ulcers, which have been rarely reported in previous studies. Her symptoms may be related to the weakening of HH signaling caused by the *DHH* variant. However, these findings need to be further confirmed by long-term follow-up of more cases and further basic research.

## Conclusion

In summary, we reported a case of 46, XY GD with a *DHH* homozygous variant resulting in a complete female phenotype, characterized by amenorrhea with neuropathy. The symptoms of peripheral neuropathy were not typical at first but worsened during our follow-up. For the first time in such patients, we showed a comparison of NCS changes with age as well as the presence of multiple complications. Our report expanded the phenotypic spectrum of diseases caused by *DHH* variants, and our long-term follow-up and summary of the literature would contribute to the future diagnosis and treatment of individuals with *DHH* variants.

## Data Availability

The original contributions presented in the study are included in the article/Supplementary Material; further inquiries can be directed to the corresponding author.
