# Supplementary material for: Case Report: Long-term follow-up of desert hedgehog variant caused 46, XY gonadal dysgenesis with multiple complications in a Chinese child
Source: Front Genet. 2022 Aug 22;13:954288. doi: 10.3389/fgene.2022.954288 (PMC9441908; doi:10.3389/fgene.2022.954288)
Supplement: Supplementary file 1 [file DataSheet1.DOCX]

**Supplementary Table 1.** Analyzed genes of 64 genes related to DSD

| Analyzed genes |
| --- |
| *AMH、AMHR2、AR、ARL6、ARX、ATRX、BBS1、BBS10、BBS12、BBS2、BBS4、BBS5、BBS7、BBS9、CBX2、CCDC28B、CEP290、CHD7、CYP11A1、CYP11B1、CYP17A1、CYP19A1、CYP21A2、DHCR7、DHH、DMRT1、FGF8、FGFR1、GNRH1、GNRHR、HS6ST1、HSD17B3、HSD3B2、KAL1、KDM6A、KISS1、KMT2D、LHCGR、LZTFL1、MAMLD1、MKKS、MKS1、NR0B1、NR5A1、NSMF、POR、PROK2、PROKR2、RSPO1、SDCCAG8、SEMA3A、SOX9、SRD5A2、SRY、STAR、TAC3、TACR3、TMEM67、TRIM32、TSPYL1、TTC8、WDPCP、WDR11、WT1* |

Genetic analysis: After obtaining informed consent, genetic analysis of the proband and her parents was performed by Guangzhou KingMed Medical Diagnostics Center. Next-generation sequencing was utilized to sequence the exon coding regions of 64 DSD-related genes (see Supplementary Material). Variants with minor allele frequencies of < 0.05 in population databases (such as ClinVar, ESP6500, 1000genomics, dbSNP and UniProt) that were expected to affect protein coding/splicing or present in the Human Gene Mutation Database (HGMD) were included in the analysis. Missense variants were analyzed using PolyPhen2, SIFT, LRT, MutationTaster,, GERP and other software, and splice variants were analyzed using NetGene2 Server and AUGUSTUS. The candidate gene was also verified by Sanger sequencing and parental sequencing. Finally, we identified a homozygous variant in the *DHH* exon 3 (c.1027T>C, p. Cys343Arg), and a heterozygous variant was found at this site in both her parents(see Supplementary Material). According to the 2015 ACMG guidelines, this missense variant was pathogenic.


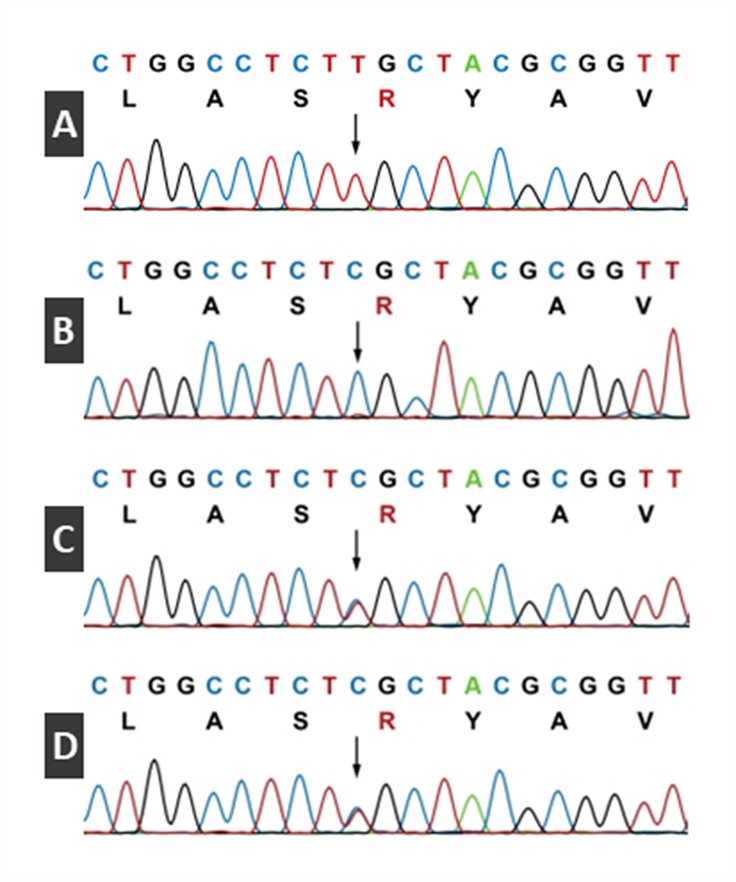


**Supplementary Figure 1.** Sanger sequencing electropherograms with mutation sites highlighted

Note: (A) Wild type, 1027T; (B) Gene sequencing results of the proband，c.1027T＞C，p.( Cys343Arg）homozygous variant; C and D represented the gene sequencing results of the parents (C for paternal and D for maternal), c.1027T＞C（p. Cys343Arg）heterozygous variant.
